# Gene-transcription factor regulatory networks implicate primary cilia in the evolution of vertebrate sex determination and expand models of epigenetic regulation

**DOI:** 10.1371/journal.pone.0353280

**Published:** 2026-07-29

**Authors:** Thea B. Gessler, Dean C. Adams, Nicole Valenzuela

**Affiliations:** 1 Department of Ecology, Evolution, and Organismal Biology, Iowa State University, Ames, Iowa, United States of America; 2 Genetics and Genomics Program, Iowa State University, Ames, Iowa, United States of America; Leibniz Institute on Aging - Fritz Lipmann Institute (FLI), GERMANY

## Abstract

The molecular architecture underlying diverse vertebrate sex-determining systems remains elusive despite fragmentary evidence of changes in upstream regulators and downstream mediators. Here we modeled species-specific regulatory networks of urogonadal development for turtles with contrasting mechanisms [*Apalone spinifera –* ZZ/ZW genotypic sex determination (GSD)*,* and *Chrysemys picta* – temperature-dependent sex determination (TSD)] using matched data from time-course sampling. We uncovered key steps in the evolutionary transition of sex determination by testing for conservation or divergence of network modular components. Specifically, we tested these alternative hypotheses: first, transcription factor (TF) hubs and their target genes are conserved between species (null H0); second, the same TF hub acquired a new set of target genes in a species, retaining or not ancestral functions (H1 and variants); third, a new TF hub took over the regulation of the former gene targets of an ancestral TF (H2); and finally, complete overhaul occured where both ancestral TF hubs and their target genes were replaced in a species (H3). Results implicate primary cilia as integrators of environmental signals underlying TSD, because known thermosensitive TSD components (e.g., calcium-redox, pSTAT3, *Wnt*/*Rspo1*/*β-catenin*, *Dhh*) overrepresented in our results are linked to primary cilia. TFs that evolved between species also regulate primary cilia and point to key changes in their sensory machinery that accompanied TSD-GSD transitions (e.g., calcium/ion channels or membrane transport components in *Chrysemys* versus structural elements and ciliogenesis in *Apalone*). This novel Primary Cilia Integration hypothesis expands current models of epigenetic regulation of turtle sexual development, the evolution of plasticity versus canalization, and warrants functional validation.

## Introduction

Vertebrate sex determination provides a compelling example of a developmental mechanism that diverges greatly despite sharing highly conserved gene players [1–4], a case of developmental systems drift [5]. Specifically, vertebrate sex-determining mechanisms (SDMs) span a spectrum from fairly strict environmental sex determination (ESD) to strongly canalized genotypic sex determination (GSD) [6,7]. SDMs are governed by genes recycled frequently among taxa, but their position or regulation in the network is shuffled repeatedly by evolution, such as occurred for *Dmrt1* [8–10], *Sox9* [11], and *Aromatase* [12], among many others. Importantly, our full understanding of the rewiring of the genetic network underlying the diversity of vertebrate SDMs is obscured in part because much of our knowledge still relies on mouse and human models [3].

Turtles are a vertebrate group particularly suited to unravel developmental network divergence as they showcase temperature-dependent sex determination (TSD)—a type of ESD, plus XX/XY and ZZ/ZW sex chromosomal systems of GSD [13]. These systems evolved independently multiple times, accompanied by accelerated molecular evolution of sexual development genes [14–16]. These natural experiments enable comparative analyses between closely related lineages with and without plastic sexual development to uncover the shifts in molecular architecture underlying these transitions [15,17] aided by growing ’omic resources in turtles [18–20]. Active research is devoted to identifying the elusive environmental sensor(s) that distinguishes TSD from GSD. Recent potential candidates include calcium and redox (CaRe) status sensors [21], and epigenetic histone modifications driving the temperature-specific activation or repression of *Dmrt1* [9,22], a masculinizing gene also implicated in a GSD turtle [23]. However, broader changes have accrued between TSD and GSD networks beyond these few candidate elements. Comparisons across turtles and other vertebrates revealed that transcriptional heterochrony contributes to the divergence in SDMs [1,6,24,25], such that agnostic genome-wide investigations should substantially illuminate these evolutionary events more broadly.

Divergence of developmental programs underlying phenotypic evolution often involve the evolution of gene regulatory network (GRN) components, such as cis-regulatory elements (CREs) that change the interaction between transcription factors (TFs) and the genes they regulate [26]. This is true for GRNs controlling animal sexual development, which at a broad taxonomic scale appear quite evolutionarily labile at the upstream trigger and more conserved in the downstream TFs [27]. TFs may regulate a multitude of genes in a GRN, acting as TF hubs, and may have pleiotropic effects in multiple biological processes. Thus, because changes in hubs can lead to profound phenotypic changes with potentially detrimental fitness effects [26], highly connected and central hubs are expected to evolve at a slower rate than peripheral elements of the GRN [28] (although adaptive evolution via changes to central hubs may be possible [28,29]), whereas changes can accumulate more gradually via shifts in gene target identity [26].

**Fig 1:**
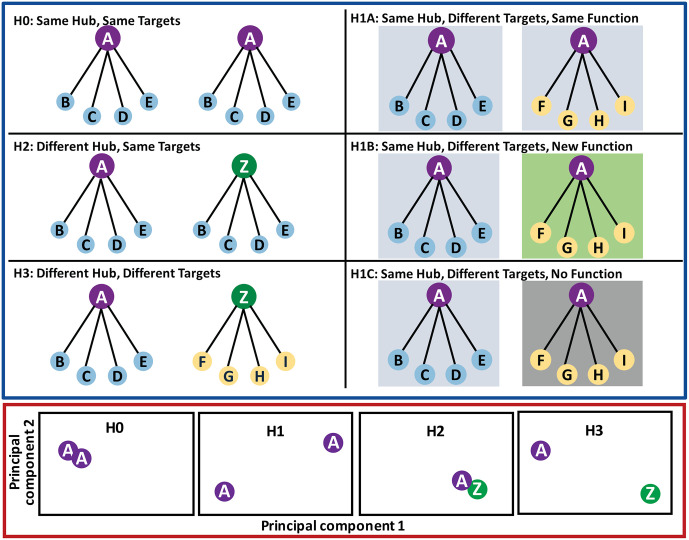
Illustration of predictions from the hypotheses of the evolution of vertebrate sex determination by shifts in the interactions of transcription factors and their predicted target genes. Top three rows encircled in blue: (H0) null hypothesis, i.e., network/module conservation, (H1) upstream conservation, downstream divergence, (H2) upstream divergence, downstream conservation, and (H3) full network/module overhaul. Bottom row encircled in red: depiction of the pattern of TF clustering in regulome space (i.e., principal components plot capturing differences in targeting patterns of TF hubs) expected under hypotheses H0, H1, H2, and H3 (see text for details).

Here we constructed and compared gene-transcription factor interaction networks for two turtle species with contrasting SDMs: the painted turtle *Chrysemys picta*, a TSD representative, and the spiny softshell turtle *Apalone spinifera*, a GSD species with an evolutionarily derived ZZ/ZW mechanism [16,30,31]. Species will be referred to by their genus name hereafter. This approach allowed us to build novel models about the molecular circuitry of TSD and GSD, test their predictions of how this circuitry evolved in these distinct lineages, and identify candidate elements for a putative role as key TSD or GSD regulators. Results indicate the involvement of primary cilia in the regulation and evolution of vertebrate sex determination. Primary cilia are organelles that nearly all cell types use to sense the context of cues from the external environment or internal signaling pathways [32]. Specifically, we tested the following non-mutually exclusive hypotheses about evolutionary patterns of turtle sexual development networks, by examining differences between species in TF hubs (i.e., TFs regulating many genes in the network), their target genes (Fig 1), and shifts in the associated functional annotations of candidate genes that would suggest a potential change in their regulatory role (i.e., a putative change in function). Of note, we use the terms hub and TF interchangeably, as the TFs examined here act as hubs within the subnetworks of the genes they target (the focus of much of our comparisons), while recognizing that in the context of broader networks some TFs may be more interconnected (i.e., hub-like) than others:

**H0: Same hub, same targets hypothesis** Neither the hub TFs nor their target genes vary between species. If this null hypothesis is supported, it would indicate evolutionary conservation of the network wiring between TSD and GSD. Partial support of this hypothesis for only some TFs would expose network connections under strong stabilizing selection. In contrast, such a finding for the entire network would be surprising given that these taxa split 175 Mya (timetree.org) and *Apalone*’s softshell turtle family shifted from TSD to GSD [31].

**H1A: Same hub directs same function with new targets** A conserved TF hub recruits a novel set of target genes that take over the regulation of the ancestral function. Support for H1A would uncover TFs subject to developmental systems drift [5] because the TF continues to direct the same function (determined by finding a conserved functional annotation of the targets in an overrepresentation test), but the gene targets implementing that function have changed between species.

**H1B: Same hub directs different function with new targets:** A conserved TF hub recruits a novel set of target genes, and these targets regulate a new function. Such a change could occur *via* natural selection for a new function via the rewiring of the TF’s target genes (in which case an overrepresentation test would reveal species-specific functional annotations for each set of gene targets).

**H1C: Same hub, new targets with no function:** The TF hub remains conserved, but it regulates new target genes that have no detectable functional annotation as determined via overrepresentation tests (i.e., potential pseudofunctionalization). This pattern could result from genetic drift, perhaps when the original function regulated by a TF is released from selection by changes elsewhere in the network, facilitating gains or losses of TF binding sites (TFBSs) irrespective of their functional potential.

**H2: New hub, same targets.** A new TF hub takes over the regulation of ancestral gene targets. If supported, H2 would uncover the evolution of upstream regulation of network modules underlying sexual development that could occur by natural selection or developmental systems drift. This would be supported by finding a similar set of regulatory targets and functional annotations for non-orthologous TFs between species.

**H3: New hub, different targets.** H3 implies the overhaul of the sexual development network either in its entirety, or for some of its modules, and would be supported if major differences are found in the identity of TF hubs, their connectivity, and their target genes, which could be carrying out either the same function as the ancestral hub and targets, a new function (neofunctionalization), or no function (pseudofunctionalization).

## Materials and methods

We constructed models of gene-TF interaction networks for both *Chrysemys* and *Apalone* using PANDA as implemented in the R package pandaR [33,34]. As input to construct the networks, pandaR takes gene expression data, TF binding site data, and protein-protein interaction data. PANDA utilizes a message passing algorithm to integrate and find agreement among multiple datasets, *via* similarity calculations, building a consensus network with predicted regulatory relationships [33]. We describe the generation of each of those datasets below.

### Gene expression dataset

We used RNA-seq data from a previous study [19], which we generated by incubating eggs from both *Chrysemys* and *Apalone* turtles at temperatures that are either 100% male- or female-producing in *Chrysemys* (MPT: 26°C and FPT: 31°C, respectively) following our standard protocols [35]. These temperatures are within the optimal developmental range for both species. Tissue was collected from both species across five matched stages of development [sensu a common staging table [36], instead of the species-specific criteria [37,38] and included the following tissues: stage 9 – trunks, stages 12 and 15 – adrenal kidney gonad complexes (AKGs), and stages 19 and 22 – gonads. Stages 9 and 12 precede the thermosensitive sex determination period (TSP) in *Chrysemys*, stage 15 sits at the onset of the TSP, whereas stages 19 and 22 are at the mid and late TSP. This sampling at stages 9−15 accounts for potential thermal effects on sex ratios prior to the canonical thermosensitive period detected in TSD turtles [39,40], encompasses the formation of the adrenogonadal primordium, and captures contributions from the earlier mesonephros to the developing gonad [41]. We note that sexual differentiation in *Apalone* takes place at similar stages as in TSD turtles [42]. Embryos of *Chrysemys* were presumed to be developing males or females according to their incubation temperature, while *Apalone* embryos were sexed by PCR using molecular markers [43], an improved sexing technique compared to qPCR of rRNA genes [44]. Thus, *Apalone* samples correspond to a full factorial dataset with male and female embryos developing under temperatures that represent the ancestral MPT and ancestral FPT. Two biological replicates (RNA libraries) were generated for each condition (species by stage by temperature, and by sex for *Apalone*), and each library included RNA pooled from 11−15 embryos. At least 40 M clean Illumina 150 bp paired-end reads were generated per library (with a 94–97% retention rate per library). Further details of the transcriptomic datasets were previously reported [19]. Reads were trimmed with Trimmomatic (v0.36) [45] and mapped with GSNAP (v20170317) [46,47] to a reference genome for each species: *Chrysemys* GCF_000241765.3_Chrysemys_picta_bellii-3.0.3 [18] and *Apalone* BioProject: PRJNA837702 [48]. Using StringTie (v1.3.4) [49], reads were assembled into transcripts by library, merged, and their abundance was calculated. Then, transcripts and counts were consolidated into gene models with tximport (v1.10.1) [50]. Finally, counts were TMM-normalized to correct for library size and log_2_-transformed to correct for heteroskedasticity using EdgeR (v3.24.3) [51].

### Transcription factor binding site (TFBS) motif dataset

Promoter sequences, defined as −1.5Kb and +500 bp surrounding the transcription start site, were extracted for all annotated genes from the *Chrysemys* and *Apalone* reference genomes. While DNA sequence can turnover rapidly, DNA binding domains for TFs are highly conserved in vertebrates and recognize the same binding motifs across lineages [52–54]. Given this documented conservation, we used vertebrate TF binding matrices from the open access JASPAR Core Vertebrate database (v2020) [55]. TF position weight matrices were searched against these promoter sequences, using CiiiDER (v1.10.6) [56] with the deficit parameter set to a highly stringent 0.05 to create a map indicating whether promoters contained putative binding sites for these TFs. Our approach centers the search for TFBS motifs around promoter sequences, permitting the identification of putative binding sites, as such sequences could affect gene regulation irrespective of their evolutionary origin [57–60].

### Protein interaction dataset

Protein sequences and protein-protein interaction data from vertebrates were obtained from the STRING database (v11.0) [61] which included data from 41 vertebrates (30 mammals, 3 birds/reptiles, 1 amphibian, and 7 fishes). In parallel, we obtained the longest translated CDS for proteins present in the *Chrysemys* genome [18]. Because PANDA focuses on gene-TF interactions, the list of *Chrysemys* proteins was filtered down to retain TFs also present in the JASPAR core vertebrate database (v2020). Because the data on protein-protein interactions draws upon other vertebrate species, we employed stringent filtering to increase the likelihood that results would be conserved in turtles. Using a reciprocal best blast approach (blastp), we compared the sequences of the STRING interacting proteins to the subset of *Chrysemys* TFs and did a final filtering step resulting in a list of TFs with a percent identity of ≥90% and resulting query coverage of ≥99%. STRING proteins and their interactors that passed through these filters were retained and assumed to represent protein interactions likely present in *Chrysemys* due to their high level of homology. For *Apalone*, protein sequences from *Chrysemys* representing putative interacting proteins were searched (tblastn) against the *Apalone* genome [48] and their presence was confirmed, such that the same protein interaction file was used as input for both species.

### Building models of gene – transcription factor interaction networks with PANDA

For *Chrysemys*, each gene expression dataset per temperature (26 and 31°C) consisted of ten gene expression libraries, encompassing two biological replicates from five developmental stages, yielding 20 libraries total. These temperature-specific libraries were used as input to predict MPT- and FPT-networks in PANDA (Cpi-Female-31°C network and Cpi-Male-26°C network). Because each incubation temperature produced both sexes in *Apalone*, there were 40 datasets for this GSD species, i.e., two replicates per stage for each sex-by-temperature combination. Sex-by-temperature sets were used as input in PANDA to generate 4 networks (Asp-Female-26°C, Asp-Female-31°C, Asp-Male-26°C, and Asp-Male-31°C). We will refer to these networks as Cpi-FPT, Cpi-MPT, Asp-F26, Asp-M26, Asp-F31, and Asp-M31 hereafter.

The PANDA algorithm was implemented with the R package pandaR (v1.22.0) [34], using as input the gene expression, TFBS motif, and protein-protein interaction datasets described above. Default settings were used in most cases, but the mode was set to ‘intersection’ to only include TFs present in the gene expression dataset while keeping network size to a manageable scale. Following network construction, it was discovered that the mode option works differently than described in the pandaR manual, so we manually confirmed which TFs in the final network were expressed in our transcriptomes and disregarded unexpressed TFs when interpreting the results. Networks produced by PANDA consist of gene-by-TF matrices populated with similarity scores (akin to Z scores) describing the likelihood that an edge (i.e., the connection between a gene and TF that defines a regulatory relationship) is true, where positive values represent greater support for an edge and negative values represent lesser likelihood that an edge is true.

**Fig 2:**
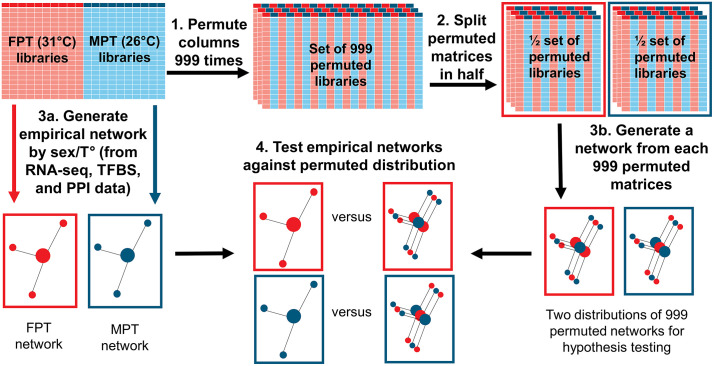
Pipeline of the permutation procedure used to generate random distributions for network comparisons using the *Chrysemys* data as an example. First, a matrix containing normalized gene expression values for all 20 libraries (red columns = FPT/female libraries, blue columns = MPT/male libraries) were permuted 999 times to generate 999 matrices with random column order. Second, each of these 999 matrices were split in half. Third, each 10-column set of submatrices was used as input to generate a random distribution of networks. Fourth, each empirical network derived from the original libraries was tested against one of the random distributions. The same process was used for *Apalone*’s 40 libraries for *Apalone* as detailed in the text. TFBS = Transcription Factor Binding Site; PPI = Protein-Protein Interaction.

To test for differences between networks, we used a permutation approach implemented with a custom R script to build a test distribution of networks (Fig 2). For this, we first built a gene (row) by library (column) matrix populated with gene expression values (20 columns in the case of *Chrysemys*). Then, we ran 999 permutations randomizing the order of the libraries (columns) in the matrix. The resulting 999 permutated matrices were split in half (columns 1–10, columns 11–20 for *Chrysemys*) to generate two sets of 999 matrices with permuted columns, and a network was generated (as described above) from each set to obtain two random distributions of ‘permuted’ networks against which to test each empirical network. The empirical FPT network was tested against one distribution while the empirical MPT network was tested against the second distribution. Likewise, for *Apalone*, the initial 40-column matrix (for the 40 libraries) was also permuted 999 times, and these randomized matrices were split in four groups (columns 1–10, 11–20, 21–30, 31–40) to generate four sets of 999 permuted matrices. A network was generated from each set of permuted matrices in PANDA to obtain four random distributions against which to test each empirical network (Asp-F26, Asp-M26, Asp-F31, and Asp-M31).

### Analysis of differential gene – transcription factor networks

We conducted within-species comparisons of gene-TF networks which tested for differences between temperatures in *Chrysemys* (Cpi-FPT vs Cpi-MPT), while for *Apalone*, comparisons tested for differences between-sex per-temperature (Asp-F26 vs Asp-M26, Asp-F31 vs Asp-M31), and between-temperatures per-sex (Asp-F26 vs Asp-F31, Asp-M26 vs Asp-M31).

Networks consist of nodes connected by edges. An edge is a vertex connecting a gene to a TF, representing the presence of an interaction. We tested for edge weights that were significantly different between two networks of interest. Using a t-test, the difference between each predicted edge (e.g., network *i* edge minus network *ii* edge) was compared to the average difference for that edge between all permuted pairs of networks. Significance was assessed following a Benjamini-Hochberg correction for multiple comparisons.

In addition to examining differential network edges, we also examined differences in network targeting patterns, of which there are two types [62,63]: gene in-degree and TF out-degree. Gene in-degree describes how many edges in the network point to a gene, while TF out-degree, describes how many edges point from a TF to various genes. Thus, these values help identify which genes and TFs of the networks are highly connected, and whether differences in the in-degree and out-degree patterns exist. In-degree and out-degree values were calculated with the pandaR function calcDegree() which sums edge weights for a particular vector (i.e., the row of TFs targeting one gene, or the columns of genes targeted by one TF). Similarly, for the differential edge calculation, a t-test was performed to assess differences in targeting patterns between pairs of empirical networks relative to the test distribution of differences in targeting patterns for all pairs of networks. Significance was assessed following a Benjamini-Hochberg correction for multiple comparisons.

We also assessed overall differences in the gene:TF networks by calculating the sum of squares of each network’s matrix and comparing the difference of the sum of squares between empirical networks to the distribution generated by calculating the differences in the sums of squares of all pairs of permuted networks. Thus, we assessed networks for differences at the edge (individual gene-by-TF targeting patterns), vector (TF regulatory patterns for collective gene targets), and matrix (whole network regulatory patterns) levels.

### Principal components analysis and trajectory analysis

We carried out a modified trajectory analysis [64] to test for differences in the regulatory targeting patterns of orthologous TFs. For this, we first filtered all six networks (2 from *Chrysemys* and 4 from *Apalone*) to identify the set of genes and TFs shared between species and transformed negative edge-weights to zero to denote a lack of relationship. Principal components analysis was then performed on the edge weights representing the genes targeted by each TF. The resulting principal components plot where the differences in targeting patterns of TF hubs are visualized constitutes the “regulome space”. These networks were further filtered to only include TFs with an average expression level of >1.5 log_2_ (TPM) across all libraries in the initial input gene expression data (to rule out negligibly expressed TFs) and that were present in all 6 networks. Next, the principal components were subjected to the modified trajectory analysis [64], which connects centroids of orthologous TFs by a vector in regulome space. The centroid is the position of a TF in the regulome space informed by the genes it regulates in the networks within each species (and is calculated from each set of species-specific networks), and the distance between centroids measures how similar or different TFs are relative to one another in the genes they are predicted to regulate between species. Longer vectors denote greater divergence in the gene targets of ortholog TFs between species, compared to TFs connected by shorter vectors in the PC Euclidian plane. To measure these distances between species for each TF, we modeled gene targets as a function of TF, species, and their interaction (linear model: Gene Targets ~ TF + Species + TF: Species). Distances between the centroids of the principal components of the target genes for a particular TF per species were calculated. We then generated a distribution of distances under the null hypothesis that there were no differences between species (no effect of species on TF). This corresponds to the prediction that no evolution has occurred in the regulatory role of these orthologous TFs since *Chrysemys* and *Apalone* split ~175 mya (timetree.org) (hypothesis H0). We tested the distances of each TF between species against this distribution using a permutation test and applied a Benjamini-Hochberg correction for multiple comparisons with an exploratory FDR of 0.1 given our sampling limitations. Using this level of FDR the top ~ 5% of distances of TFs and those belonging to the right-most distribution in our bimodal distribution of distances were considered significant. Non-significant distances between orthologous TFs between species reject hypothesis H1 and may denote TF hubs conserved between lineages in their target patterns (supporting hypothesis H0), whereas orthologous TF hubs with significantly longer distances would have diverged between species in the identity of the genes they regulate (hypothesis H1) (Fig 1). We tested hypothesis H2 by measuring trajectory distances of non-orthologous pairs of TFs between species. Here, we identified interspecific trajectories that were shorter than expected under the null hypothesis, indicating non-orthologous (analogous) TFs that converged on the same gene targeting pattern between species (supporting hypothesis H2). Additionally, we confirmed that these trajectories were shorter than the distance to their respective orthologous TFs (albeit not significantly shorter since they were all above the critical value of the 5% quantile). Hypothesis H3 was tested by consilience with the other hypotheses.

### Gene ontology overrepresentation analysis of networks

The PANTHER (v17.0 – date: 2022-02-02) online GUI [65] was used to conduct overrepresentation analysis of gene targets of TFs of interest. We used a Fisher’s Exact Test with an FDR correction to determine which gene ontology (GO SLIM) terms (molecular function, cellular component, and biological process) were overrepresented in the targets [66], as well as if any PANTHER pathways [67] or PANTHER protein classes were overrepresented. Since our focal species are non-model organisms that are not represented in the PANTHER databases, we first mapped the translated CDSs from *Chrysemys* to PANTHER IDs using PANTHER HMM scoring tools (pantherScore2.2) to score our sequences against the PANTHER HMM library (v17.0), following the instructions provided by the PANTHER developers. In the case of duplicate hits due to redundancy in the *Chrysemys* genome, we prioritized the result to those with the highest bitscore. Since *Apalone* annotations were based on the *Chrysemys* genome, we used the mappings obtained from *Chrysemys* CDS sequences to transfer the corresponding annotations to genes in the *Apalone* networks. Only TFs with at least 50 known gene targets prior to uploading to PANTHER were included in the overrepresentation analysis, as functional annotation results can be sensitive to input list size [68,69]. Additionally, to focus the analysis on gene targets with the highest support in the network models, gene targets were filtered to retain those with an edge weight in the top 5% of all edge weights. Our approach resulted in sets exceeding the 50-gene benchmark, as most sets analyzed had 100s or 1000s gene targets in their input list. Tabular results of the analysis were downloaded and saved locally. The background gene set included all genes present in the network, but because not all genes mapped to panther IDs the total effective number was smaller (CPI: 16489 genes, ASP: 11386 genes).

### Transcription factor functional similarity analysis via calculation of semantic similarity

The resulting sets of overrepresented terms of TF gene targets were compared for semantic similarity with GOGO [70] to determine their degree of functional similarity. Shifts in functional annotation are suggestive of putative changes in function that require functional validation (referred to as changes in function hereafter for simplicity). GOGO is a hybrid algorithm for semantic similarity calculations that uses both the topology of the directed acyclic graphs (DAG) that make up the ontology (which informs ancestor-child term relationships) and considers the number of children nodes of a term (which reveals the information contained in a term). Thus, it allows us to impartially assess how similar two lists of gene ontology terms are to one another which can be hindered by their hierarchical nature. We updated the DAGs used by the program to match the same reference ontologies used to run the overrepresentation tests (PANTHER v17.0). We used the gene_list_comb.pl script to calculate semantic similarity of the sets of statistically significant GO terms returned for the gene targets of a TF in a particular network. We then compared the similarity scores, which range from zero (no similarity) to one (perfect overlap), to assess the degree of putative functional similarity of TF targets for orthologous TFs across networks. We used a Mann-Whitney U test to evaluate whether there were significant differences in semantic similarity for the set of within-species comparisons (all *Chrysemys* by *Chrysemys* and all *Apalone* by *Apalone* contrasts) relative to the set of between-species comparisons (all *Chrysemys* by *Apalone* contrasts) and applied a Bonferroni correction for multiple comparisons.

### Subnetworks

Because the final networks obtained from PANDA for *Chrysemys* contained 2,883,632 edges, we also generated subnetworks to focus our attention on the most strongly supported edges, by filtering networks for the edges with an arbitrary selected Z score > 10 (which corresponds to the top 0.03% of edges for *Chrysemys* and *Apalone*). Subnetworks were visualized in Cytoscape (v3.9.0) [71], and the hubs and their targets were identified using the function targetedGenes() for overrepresentation analysis of gene ontology functional terms.

All scripts used for the analyses are included in the Supporting Information.

## Results

### Putative core network components of turtle sexual development

Models of gene-TF interaction networks were constructed using PANDA [33,34] with matched-stage RNA-seq data from a previous study of *Chrysemys* and *Apalone* embryos [19] (trunks at stage 9, adrenal kidney gonad complexes at stages 12 and 15, and gonads at stages 19 and 22), transcription factor binding sites from their reference genomes [18,48], and vertebrate protein-protein interaction data [61], which yielded two networks for *Chrysemys* (Cpi-Female-31°C and Cpi-Male-26°C), and four networks for *Apalone* (Asp-Female-26°C, Asp-Female-31°C, Asp-Male-26°C, and Asp-Male-31°C). We will refer to these networks as Cpi-FPT, Cpi-MPT, Asp-F26, Asp-M26, Asp-F31, and Asp-M31 hereafter. For *Chrysemys* and *Apalone*, all network pairs within species were nearly identical when analyzed for differential edges (an edge is a network vertex connecting a gene to a TF, representing the presence of an interaction), differential targeting patterns (i.e., gene in-degree and TF out-degree patterns, which describe how many edges point to a gene, and how many edges point from a TF to various genes, respectively), and overall network differences at the full matrix level (i.e., *Chrysemys*: Cpi*-*MPT vs Cpi-FPT; *Apalone*: Asp-M26 vs Asp-F26, Asp-M31 vs Asp-F31, Asp-M26 vs Asp-M31, and Asp-F26 vs Asp-F31). Namely, no differential edges or differences in targeting patterns were detected after Benjamini-Hochberg correction, and the sum of squares calculation assessing overall network differences was also non-significant (p > 0.2 in all cases, Table 1). The same was true if networks were built based on only differentially expressed genes. This overall similarity among networks at the global level is likely due to the small size of the dataset and the pooling of gene expression data from five developmental stages. This caveat implies that the results described here are conservative, as they represent the strongest signals that stand out despite our sampling limitation. The coarse global comparison approach identified potentially evolutionarily conserved developmental signals common to all conditions and embryonic stages (not only within but also between species as described below) that may represent presumptive core components of turtle developmental processes to be functionally validated in future studies or ruled out if larger sampling uncovers differences that passed undetected here. On the other hand, the subtle sex- or temperature-specific differences that were too weak to be detected with this initial global method (masked by the broad overall similarities), were revealed by quantitative hypothesis testing. This included 89 out of 148 turtle TFs from the full networks that were expressed in the time-course transcriptomes of both *Chrysemys* and *Apalone* and thus permitted further evolutionary analyses between species.

**Table 1:** Sum of squares result from the overall network comparison.

Network Comparison	Sum of Squares	Pvalue
*Chrysemys* 26°C vs 31°C	330214.43	0.677
*Apalone* Female 26°C vs Male 26°C	226941.63	0.376
*Apalone* Female 31°C vs Male 31°C	264538.08	0.224
*Apalone* Female 26°C vs Female 31°C	146894.62	0.853
*Apalone* Male 26°C vs Male 31°C	237478.87	0.329

### Principal components and trajectory analysis

**Fig 3:**
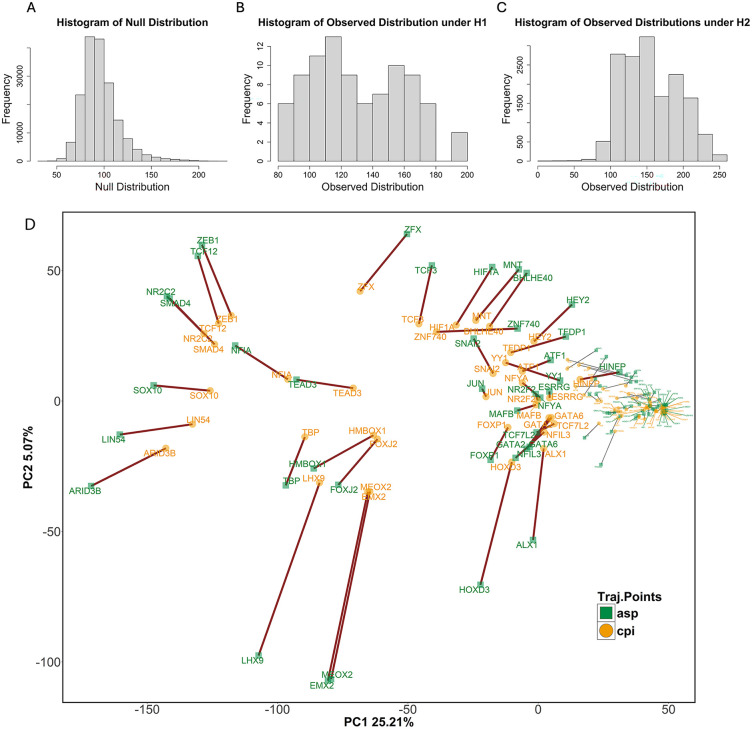
Trajectory analysis of the evolution of 89 transcription factors between *Chrysemys* and *Apalone.* Null (A) and observed (B and C) empirical distributions of distances (trajectory magnitudes) between orthologous TFs (B) and non-orthologous TFs (C) in regulome space (D). Trajectory magnitudes are based on the edge weights between TFs and their gene targets which are represented as effect sizes (Z scores). The distribution in panel B was used to test hypothesis H1, i.e., whether trajectories between orthologous TFs were longer than expected under the null hypothesis H0 (that no evolution occurred between these two turtle lineages over 175 my). The distribution in panel C was used to test hypothesis H2, i.e., whether trajectories between non-orthologous TFs were shorter than expected under the null hypothesis H0, which would indicate that non-orthologous TFs converged between species on a common set of gene targets. (D) Greater divergence between species in the gene targeting patterns of orthologous TFs is denoted by longer vectors (greater Euclidean distance in the principal components regulome space) between *Apalone* and *Chrysemys*. Thicker brown lines indicate significantly longer trajectories than expected, whereas thinner gray lines indicate trajectories of length expected by chance (supporting H0).

We employed principal components (PC) analysis on the edge weights representing the genes targeted by each TF to more clearly discern the patterns present in these hyperdimensional ‘omics data, after retaining only genes and TFs shared between species across all six networks, transforming negative edge-weights to zero to denote a lack of relationship, and excluding negligibly expressed TFs with average expression <1.5 log_2_(TPM) [TPM = transcripts per million]. Next, the principal components were subjected to a modified multivariate trajectory analysis [64] to test for differences in the regulatory targeting patterns of orthologous TFs. We compared the length of the vector that connects centroids of orthologous TFs in regulome space to the length predicted under the null hypothesis (H0) that there were no differences between species (Fig 3A-C). Regulome space provides a measure of how similar or different TFs are relative to one another in the genes they are predicted to regulate between species. PC1 and PC2 captured 25.2 and 5.1% of the variation, respectively (Fig 3D), while 888 PCs explained all the variance, reflecting the high complexity of these networks and the many factors that contribute to their variation.

Results from the multivariate trajectory analysis on this principal components’ space (the regulome space) revealed 50 of 89 TFs that supported the null hypothesis H0 (same hub, same targets) as they were conserved in their regulatory targets (Fig 3A). Hypothesis H1 (same hub, different targets) (Fig 3B) was supported by the remaining 39 TFs that significantly diverged from their ortholog in the identity of genes targeted or in the strength of their connection (Fig 3D). Next, we conducted overrepresentation tests of the functional annotations of the predicted regulatory targets of these 39 TFs obtained using The Gene Ontology and calculated the similarity between orthologs using semantic similarity (see methods for details). We found 5 TFs (ARID3B, EMX2, LHX9, LIN54, and MEOX2) exhibited significant overrepresentation test results across all 6 networks (FDR of 0.05), enabling full cross-species comparison of their semantic similarity for hypotheses testing of the three alternative explanations of their divergence in regulome space (Table 2). Semantic similarity considers the gene ontology terms and graph structure to assess differences in functional annotation (i.e., putative functional role) [70]. We analyzed these 5 TFs to determine whether the functional role of their new targets remained conserved despite the turnover in target identity (hypothesis: H1A), whether a new functional role was acquired (hypothesis H1B), or whether a clear function was lost (hypothesis H1C) (Fig 1), and found ARID3B supported H1A, while EMX2, LHX9, LIN54, and MEOX2 supported H1B. Potential support for hypothesis H2 that a non-orthologous TF took over the regulation of an ancestral TF hub, was found for a single TF in the GSD *Apalone* (ZBED1) that is closer in regulome space than expected under the null H0 to 12 TFs in the TSD *Chrysemys* (BACH2, BCL6, CTCF, GLI2, GLIS3, IRF1, MTF1, NR2F6, PKNOX2, RXRA, TGIF1, ZNF410). Intriguingly, the ZBED1 ortholog was not significantly farther apart than expected (failed to reject H0), thus, ortholog targeting patterns did not differ between species. Combined, these results suggests that either *Apalone* ZBED1 acquired a new regulatory role without giving up its ancestral regulatory role, or alternatively, that the 12 TFs in *Chrysemys* slightly converged on ZEBD1’s targets. Lastly, while the biologically-relevant analysis includes the 4 networks from *Apalone* because both sexes developed at each incubation temperature in this GSD species, we carried out a sensitivity test by rerunning the trajectory analysis using both *Chrysemys* networks and pairs of male and female *Apalone* networks as listed in Table 1, and found that results are qualitatively robust (the same 39 TFs remained ranked in the top 39 positions, and the top 37 retained significant unadjusted p-values). We thus restrict the follow up discussion to these 37 TFs.

**Table 2:** Semantic similarity in biological process (BP), cellular component (CC) and molecular function (MF) for transcription factors that evolved significant differences in targeting patterns between *Chrysemys* and *Apalone* as determined by trajectory analysis of the principal components regulome space.

Transcription Factor	Trajectory Analysis *P* value	Semantic Similarity Differences Between Species *P* value	Between Species Gene Overlap	Within CPI Gene Overlap	Within ASP Gene Overlap
ARID3B	0.072	BP, CC, and MF: all NS	9.3-11.6%	44.7%	53.7-67.3%
EMX2	0.054	**BP: 0.022**	3.9-5.7%	51.3%	36.4-52.8%
LHX9	0.087	BP: NS; **CC: 0.014**	4.9-6.8%	49.0%	39.6-53.5%
LIN54	0.072	**BP: 0.037; CC: 0.025**	5.7-7.5%	39.5%	41.8-53.5%
MEOX2	0.042	**BP: 0.015;** CC: NS	3.6-5.7%	50.5%	34.9-52.2%

Semantic similarity indicates how similar the sets of gene ontology terms annotated to a TF were when compared between species while accounting for hierarchical graph structure. P-values are provided for TFs with significant differences in their semantic similarity between species at an exploratory α = 0.1 which corresponded to the top 5% of TFs that occupied the upper mode of the observed distribution (Fig 3B). The degree of overlap in the sets of genes regulated by each TF is also provided, including: the range of gene overlap for all *Chrysemys* vs all *Apalone* (Between Species), *Chrysemys* MPT vs *Chrysemys* FPT (Within CPI), and the range of gene overlap for all *Apalone* vs all *Apalone* (Within ASP) to illustrate the degree of change in gene targeting patterns between species. See the Supporting Information for the full list of overrepresentation results. NS = Non-significant.

### New targets of ARID3B, a gene linked to primary cilia sensory mechanism, carry out a conserved function in support of hypothesis H1A

ARID3B, a factor expressed in the Leydig cells of human and mouse testis [72] that affects the expression of *Wnt1* and other genes in the placenta [73], was highly and constitutively expressed throughout development in both turtle species and both sexes [19]. Here, ARID3B was more distant in regulome space between species than expected under the null hypothesis (H0), and relatively low overlap was detected in the identity of ARID3B's gene targets between species (Table 2). Yet, no significant differences in semantic similarity were detected when contrasting between versus within species comparisons, indicating a conservation of ARID3B's function despite a change in gene targets (hypothesis H1A). This observation supports the notion that developmental systems drift affected the evolution of ARID3B’s gene targeting pattern between species.

When examining individual contrasts, semantic similarity of ARID3B targets was much lower between sexes in *Chrysemys* relative to within-*Apalone* contrasts, particularly for molecular function. Specifically, ARID3B targets at Cpi-FPT related to channel activity and membrane transport, which could implicate ARID3B in the epigenetic regulation of TSD female development [21,22], but mostly to cytoskeletal protein binding (tightly linked to primary cilia formation and maintenance) at Cpi-MPT. Notably, ARID3B terms relate directly to primary cilia for males incubated at 26°C in both species.

### EMX2, LHX9, LIN54, and MEOX2 are linked to primary cilia and support hypothesis H1B (same hub directs new function with different targets)

These TFs differed significantly more between *Chrysemys* and *Apalone* in their targeting patterns than expected under the null hypothesis H0 (Table 2), reflecting an evolutionary change in their regulatory subnetworks. Furthermore, they showed significant differences in functional annotation when testing for semantic similarity. EMX2 and MEOX2 showed a significant difference in semantic similarity for the biological process ontology, LHX9 for the cellular component ontology, LIN54 for both, and all showed changes in function that accompanied the change in gene targets between species (Table 2), supporting hypothesis H1B (retention of the TF hub while gene targets changed in identity and function). Notably, all four TFs returned overrepresentation terms for primary cilia. LIN54 is also related to Wnt signaling which primary cilia help sense [74]. Additionally, all four TFs diverge somewhat among sex/temperature within-species networks (Table 2), revealing a lability between sexes or species that renders them candidates of interest underlying the transition of sex determination.

**EMX2** exhibited the lowest semantic similarity between the Asp-M31 network (an ancestrally feminizing temperature) and all other *Apalone* networks. EMX2 is essential for gonadal and urogenital development in eutherian mammals [75] and a marker of the bipotential gonad early in embryogenesis [76], a time when Wnt signaling also participates [77]. This result is consistent with previous genome-wide developmental-transcriptomic trajectory analysis showing that the most sexually dimorphic gene expression in this GSD turtle occurs at the ancestrally feminizing 31°C temperature. In *Apalone, Emx2* was upregulated in females at stages 19 and 22 and downregulated in males at 31°C [19], whereas in *Chrysemys, Emx2* showed monomorphic expression [19,78]. The opposite was not true. Namely, the ancestral masculinizing temperatures (26°C) did not induce greater divergence of the Asp-F26 network as no reduced semantic similarity was detected when comparing to all other *Apalone* networks. Consistent observations were made during the between-species comparisons. That is, Asp-M31 networks returned fewer terms than all other networks, which overlapped largely with other *Apalone* networks, but not at all with *Chrysemys*, and thus showed the lowest semantic similarity with *Chrysemys* networks. Terms in Asp-M31 were related to cell projection and microtubules – possibly pointing to general primary cilia related-terms (and explicit primary cilia terms were present in all other *Apalone* networks). Meanwhile, both *Chrysemys* networks returned terms related to anatomy and multicellular development with Cpi-FPT’s terms related to the nervous system and Cpi-MPT’s terms related to transcription and the immune system (functions linked to primary cilia in other species as detailed in the discussion).

For **LHX9**, another bipotential gonad marker [76,77] important for gonadal development in turtles and mammals [79–82], the functional ontology terms of gene targets for *Apalone* were associated mostly with the cytoskeleton, which is important for various cellular structures and processes including a key role for the formation of primary cilia [83,84]. For *Chrysemys*, terms were associated with ion channels many of which reside in the ciliary membrane among multiple cellular locations. This supports LHX9’s mechanistically important role to relay temperature cues in TSD animals [20,85], and suggests the evolution of GSD in *Apalone*’s lineage may have released LHX9 from its TSD function. Consistent with this observation, the within-species cellular component semantic similarity for LHX9 was generally quite high for *Apalone* but more moderate for *Chrysemys*. This suggests the hypothesis that LHX9 is perhaps more plastic in TSD *Chrysemys* and more canalized in GSD *Apalone*. Consistently, *Lhx9* in *Chrysemys* is upregulated at MPT (26°C) throughout the thermosensitive period (stages 15, 19 and 22) whereas in *Apalone, Lhx9* retains this ancestral upregulation at 26°C throughout stages 15, 19, and 22, but it is also upregulated in stage 19 females compared to males irrespective of temperature, and in stage 22 females at 31°C [19], suggesting a putative evolutionary shift in expression and functional regulation with respect to sex, but not temperature.

**LIN54**, a factor involved in development and reproduction in the genus *Drosophila*, *Caenorhabditis elegans* [86,87], and perhaps mammals [86,88], is a core subunit of the DREAM/LINC complex that regulates DNA repair and the cell cycle [86,88–91]. The primary cilium is linked to the cell cycle as the centrosome that comprises the basal body of the primary cilium becomes the mitotic spindle of dividing cells [32,83]. LIN54 showed significant differences in semantic similarity for both biological process and cellular component between species despite showing stable expression throughout development in both sexes in *Chrysemys* and *Apalone* [19]. In *Apalone* these differences were largely driven by Asp-M26 which returned terms related to primary cilia, while other *Apalone* networks returned fewer or more general biological process terms, and cellular component terms related to the nucleus. In contrast, *Chrysemys* terms were generally related to the nervous system and to neuron and ion/cation channel and cellular periphery at Cpi-FPT, but to the immune system, Golgi-vesicle transport, muscle cell-related, cytoskeleton/microtubule, nucleolus, and ribosome terms at Cpi-MPT. Importantly, previous studies have shown that many of these components have ties to primary cilia directly or indirectly [[32] and references therein].

**MEOX2** is better known for its involvement in mesoderm differentiation [92,93] and limb development [94] but is also tied to nociception of inflammatory stimuli [95] in vertebrates. The biological process terms retrieved here for MEOX2 were strongly indicative of primary cilia and related components for *Apalone*, while *Chrysemys* networks were characterized by terms related to development for both networks with nervous system terms returned for Cpi-FPT and immune system for Cpi-MPT. Although differences in the semantic similarity of cellular component were not significant for within versus between species comparisons, the semantic similarity value between male and female *Chrysemys* was very low (0.172), unlike in *Apalone* (> 0.5 for all contrasts). This was due to the overrepresentation of calcium channel terms in Cpi-MPT but not Cpi-FPT networks, another important cellular component for relaying environmental cues during TSD gonadal development [21,22], and consistent with recent work connecting calcium to male development via aldosterone production in *Trachemys* *scripta* [96] (*Trachemys* hereafter).

### Targeted comparison of networks: subnetwork analysis also points to primary cilia

We also queried the networks qualitatively for subtler but potentially biologically important similarities and differences, focusing on hubs from highly supported subnetworks (those with edge scores Z > 10) and comparing (a) the identity of the TF hubs themselves, (b) the similarity in the identity of their gene targets, and (c) the functional annotations and overrepresentation of their gene targets. We identified 26 TFs of interest (Table 3) to further examine the molecular circuitry (network topology) of urogonadal development in *Chrysemys* and *Apalone*.

**Table 3:** Highly supported subnetwork TF hubs identified in *Chrysemys* (CPI) or *Apalone* (ASP) using an edge weight cutoff of Z > 10 (listed alphabetically).

CPI Sub-network	ASP Sub-network	Gene Name	Overrepresentation functional annotation	Trajectory results between species (H1 test)	Hypothesis supported	Reported link to primary cilia in literature
BACH2	BACH2	BTB Domain and CNC Homolog 2	NS	NS	H2	No
BCL6	BCL6	BCL6 Transcription Repressor	NS	NS	H2	No
CREB3L1	CREB3L1	CAMP Responsive Element Binding Protein 3 Like 1	NS	NS	H0	No
**CTCF**	*Z < 10*	CCCTC-Binding Factor	*CPI – no for primary cilia*	NS	H2	Yes [97]
GLI2	GLI2	GLI Family Zinc Finger 2	NS	NS	H2	Yes [98,99]
*Z < 10*	GLIS3	GLIS Family Zinc Finger 3	NS	NS	H2	Yes [100,101]
GRHL1	*GRHL1*	Grainyhead Like Transcription Factor 1	NS	NS	H0	No
*KLF13*	KLF13	KLF Transcription Factor 13	NS	Absent	*Not tested*	No
MTF1	MTF1	Metal Regulatory Transcription Factor 1	NS	NS	H2	No
MYBL1	MYBL1	MYB Proto-Oncogene Like 1	NS	NS	H0	No
NR2F6	NR2F6	Nuclear Receptor Subfamily 2 Group F Member 6	NS	NS	H2	No
PKNOX2	PKNOX2	PBX/Knotted 1 Homeobox 2	NS	NS	H2	No
PLAG1	PLAG1	PLAG1 Zinc Finger	NS	NS	H0	No
**RFX2**	**RFX2**	Regulatory Factor X2	*CPI – no for primary cilia*	NS	H0	Yes [102]
*Z < 10*	**RFX4**	Regulatory Factor X4	*CPI – yes for primary cilia*	Absent		Yes [103]
RXRA	RXRA	Retinoid X Receptor Alpha	NS	NS	H2	No
*RXRG*	RXRG	Retinoid X Receptor Gamma	NS	Absent	*Not tested*	No
SIX2	SIX2	SIX Homeobox 2	NS	Absent	*Not tested*	No
SOX11	SOX11	SRY-Box Transcription Factor 11	NS	NS	H0	Yes [104]
*SPI1*	SPI1	Spi-1 Proto-Oncogene	NS	Absent	*Not tested*	No
**TCF3**	*Z < 10*	Transcription Factor 3	*CPI – yes for primary cilia*	Sig.	H1	Yes [105]
TGIF1	TGIF1	TGFB Induced Factor Homeobox 1	NS	NS	H2	Yes [106]
**ZEB1**	*Z < 10*	Zinc Finger E-Box Binding Homeobox 1	*CPI – yes for primary cilia*	Sig.	H1	Yes [107–109]
ZIC1	ZIC1	Zic Family Member 1	NS	Absent	*Not tested*	No
**ZNF143**	**ZNF143**	Zinc Finger Protein 143	*CPI – no for primary cilia*	NS	H0	No
ZNF410	ZNF410	Zinc Finger Protein 410	NS	NS	H2	No

TFs that did not surpass the edge weight cutoff of Z > 10 in one species are denoted as *Z < 10*. *Italic font* denotes genes with low expression in one species [defined as log_2_(counts) < 1 for more than half of the libraries]. **Bold font** denotes hubs whose gene targets returned significant functional annotations in a species (detailed in the Supporting Information). Significant trajectory results indicate the gene targeting patterns diverged significantly between ortholog TFs. NS = nonsignificant; S = significant; Absent = excluded from trajectory analysis due to low expression; H0 = fail to reject hypothesis H0; H1 = evidence supports H1; H2 = evidence supports H2.

The topology of these 26 TFs and their gene target interactions in the *Chrysemys* male and female subnetworks obtained from PANDA were nearly identical to each other in their pattern of gene targeting (Table 3), and the same was true within *Apalone* subnetworks. Thus, we focused on interspecific patterns. Between species, the same set of TF hubs were highly supported in both species, yet their highest supported gene targets differed considerably between *Chrysemys* and *Apalone*. Some TF hubs in the subnetwork were lowly expressed in only one species, maybe because they experienced a loss in activity (KLF13, RXRG, SPI1 in *Chrysemys*; GRHL1 in *Apalone*). They are returned as a hub likely because TF binding sites (and thus their regulatory potential) still exist in the promoter region of ancestral gene targets, possibly because they are still active in another context (e.g., pleiotropy).

Of these 26, six subnetwork hub TFs exhibited overrepresented biological functions exclusively in *Chrysemys* (CTCF, RFX2, RFX4, TCF3, ZEB1, and ZNF143) and virtually identical annotation terms at Cpi-MPT and Cpi-FPT for each TF, suggesting their likely role in general non-dimorphic sexual development. But importantly, gene targets for each of these six TFs differed greatly between species (0.68–11.5% overlap). This low overlap between species may be due to the less complete annotation of the *Apalone* genome compared to the *Chrysemys* genome, or alternatively, it may suggest (1) a functional loss for sexual development for these TFs in *Apalone* (hypothesis H1C, pseudofunctionalization), perhaps by genetic drift and consistent with an absence of significant overrepresentation results in this GSD turtle, or (2) a change in TF regulatory roles in *Apalone* compared to *Chrysemys*, perhaps if the divergence in gene targets led to subfunctionalization (a change in regulation of a particular functional process by distributing the ancestral role across different TFs). Of note, three of these six TFs (TCF3, RFX4, and ZEB1) returned terms related to primary cilia. Moreover, RFX4 along with RFX2 play an important role in ciliogenesis, which is broadly conserved across vertebrates, and are testis regulators with RFX2 having a key role in spermatogenesis affecting ciliary and cytoskeleton remodeling genes [102,103,110–112].

Additionally, the terms related to primary cilia were overrepresented repeatedly irrespective of regulatory differences between species. Nineteen TFs returned significant overrepresentation results related to primary cilia (ARID3B, EMX2, LHX9, LIN54, and MEOX2 mentioned above, plus DLX2, HIC2, HMBOX1, HOXD3, LHX2, MSX1, NFIA, RFX4, SHOX, SHOX2, SMAD4, SOX10, TCF3, and ZEB1), of which six have documented ties to gonadal development, i.e., EMX2 and LHX9 (described earlier), plus LHX2, MSX1, SMAD4, and SOX10 [75,80,113–122]. Of note, these terms tended to be present in Cpi-MPT and Asp-M26 networks, suggesting a bias towards male development tied to cooler temperatures.

### Shifts in regulation of known sexual development genes of interest

Taking a qualitative candidate gene approach, we also queried which TFs targeted several well-known gene regulators of sexual development: *Aromatase*, *Dhh, Dmrt1*, *Nr0b1 (Dax1)*, *Nr5a1 (Sf1)*, *Sox9*, and *Wt1*. The TFs among these genes of interest lacked position weight matrices in the JASPAR 2020 database used to obtain TF binding sites data but have been studied repeatedly in *Chrysemys* and *Apalone* [19,24,25,78,123–126]. We focused on expressed TFs targeting these genes of interest and identified those whose average edge weight difference between species-specific networks was greater than 3 (a difference of 3 between networks was chosen as qualitatively indicative of substantial differences because the top 5% of edges in these networks corresponded to values greater than 3). Using this metric, TBX20 emerged as a candidate regulator of interest for *Aromatase* in *Chrysemys*, and HNF4A, IRF1, and PAX3 as regulators of interest of *Sox9* in *Apalone*, while NFIA and CTCF are putative regulators of interest for *Dhh* in *Chrysemys*. CTCF is a gene affecting 3D chromatin structure which differs between turtles and other amniotes [48] and is linked to male germline development [127,128]. TFs that showed a greater targeting pattern of *Dhh* in *Apalone* relative to *Chrysemys* were ARID3B, EMX2, LHX9, and MEOX2, identified by our trajectory analysis described earlier, plus ALX1, BARHL2, BHLHE40, ELF5, HESX1, HEY2, HIF1A, ISL2, LHX2, LHX8, MSX1, MNT, and VAX1. Many of these TFs have previously been linked to sexual development and reproduction, with some related to gonadal establishment and development [EMX2 [75,113]; LHX2 [115,116]; LHX9 [80]]; ELF5 to epididymis [129–131]; HIF1A to steroidogenesis in granulosa cells in the ovary [132]; and others to germ cell development [BARHL2 to undifferentiated spermatogonia [133,134]; LHX8 to oocyte development [135,136]; MSX1 to meiosis and germ cell migration [118,120]].

## Discussion

Extensive fragmentary data suggest that the molecular architecture of vertebrate sexual development has evolved among disparate lineages at both the upstream regulators of sex determination and at the downstream mediators of sex differentiation, recycling some genes again and again [1,4,137]. To our knowledge, our study is the first to build and compare species-specific gene-transcription factor regulatory networks of urogonadal development between two vertebrates in the same Order that possess contrasting sex-determining mechanisms, to uncover putative key steps during the evolution of sex determination in these two lineages, by testing for conservation or divergence of modular components built using data from matched time-course sampling. TF hubs identified in *Chrysemys*, a turtle that has retained the TSD condition that is ancestral to turtles were compared to their orthologs in *Apalone*, a turtle with an evolutionarily derived ZZ/ZW GSD mechanism [30,31,138], to assess the similarity of the identity and functional annotations of their gene targets. While *Chrysemys* has been used as proxy for the ancestral TSD condition in turtles in evolutionary analyses [138], we note that these turtle lineages continued evolving since their split ~175 Mya (timetree.org), such that differences between species may not be attributable solely to changes in the softshell turtle family to which *Apalone* belongs. Indeed, further research with additional taxa is warranted to fully test the directionality of the evolutionary changes inferred here.

In general, gene regulatory networks evolve by altering hubs, their targets, or the strength of their connections, and we found evidence consistent with the notion that all these processes may have been at play during the evolution of turtle sex determination. Our results from the analysis of 89 TF hubs sufficiently expressed in the transcriptomes of *Chrysemys* and *Apalone* [19] to enable testing, countered the hypothesis that the evolution of GSD required a complete overhaul of the regulatory network of sexual development (ruling out hypothesis H3, Fig 1). Instead, our findings indicated that first, most of these TFs (50 out of 89) support the null hypothesis H0 that some TFs hubs and their targets are conserved between species, perhaps representing core components of the regulatory network of turtle sexual development. Alternatively, perhaps subtle but biologically important differences in these modules passed undetected that would be revealed with more extensive sampling in the future. Second, 37 other TFs did diverge in the downstream genes they target, supporting hypothesis H1. The inspection of their putative function (assessed by their functional annotation) indicated that some of these 37 TFs retained their ancestral function (hypothesis H1A), and some gained a new function (hypothesis H1B) (discussed below). The remaining two TFs were excluded after the sensitivity test. Interpretation of H1C that TF targets lost their ancestral function is challenging as absence of evidence does not necessarily equate to evidence of absence, because lack of annotations might reflect incomplete datasets instead. Indeed, we did observe that 14 of the 37 TFs returned significant functional annotations for one species but not for the other (considering all 5 ontologies tested against, which include molecular function, cellular component, biological process, PANTHER protein class, and PANTHER pathways). Among these, there was a strong bias (13/14 cases) towards absence of functional annotations in *Apalone*, so we interpret this result cautiously, as it could be caused by the lower annotation of the *Apalone* genome compared to *Chrysemys*. Otherwise, this result would suggest an extensive pseudofunctionalization of the molecular circuitry underlying sexual development in this GSD turtle that requires further functional validation. The one case which solely returned functional annotations for *Apalone* was ESRRG, a steroid receptor, although most terms returned were general or related to RNA metabolism or gene expression. We found a single putative case of a TF (ZBED1) in the GSD *Apalone* that either took over the control of conserved targets (hypothesis H2) while retaining its ancestral function, or alternatively, of 12 TFs in *Chrysemys* that converged on ZEBD1’s targets. Overall, our findings agree with the conservation of higher order regulatory network architecture documented in eukaryotes, and that substantial divergence has accrued in the identity and function of their regulatory targets, as observed across humans, flies, and worms [139]. To date, few large comparative network studies exist, and more are needed to reveal common themes of network evolution [140]. However, mechanistic studies have added important insights to the knowledge of GRN patterns underlying evo-devo [141] and our study contributes to this active field.

### Primary cilia hypothesized to underlie sexual development and the evolution of sex determination

Numerous lines of evidence from our results suggest that primary cilia may be involved not only in TSD sexual development but also in the evolution of sex determination. **First**, five of the 37 TFs that changed targets between TSD and GSD turtles (EMX2, LHX9, LIN54, ARID3B, and MEOX2) could be functionally annotated across all six networks in turtles and showed significant results in the semantic similarity tests, enabling alternative hypotheses testing. All five returned overrepresentation terms for primary cilia, sensory organelles [32] known to be involved in mammalian urogenital development [142–144]. Our results link these organelles to turtle sexual development and to evolutionary transitions in vertebrate sex determination for the first time, to our knowledge. **Second**, our trajectory analysis identified seven other TFs (HMBOX1, HOXD3, NFIA, SMAD4, SOX10, TCF3, ZEB1) that showed significant divergence between *Chrysemys* and *Apalone*, but whose results were not comprehensive enough for hypothesis testing. Notably, these seven TFs also returned terms related to primary cilia. **Third**, several of the TFs whose regulatory functions may have been taken over at least partially by ZBED1 in *Apalone* are linked to primary cilia directly or indirectly (e.g., CTCF, GLI2, GLIS3, MTF1, NR2F6, PKNOX2, RXRA, TGIF1, ZNF410), supporting the notion that evolutionary shifts related to primary cilia accompanied the evolution of turtle sex determination. **Fourth**, ESRRG, the steroid receptor that returned functional annotations only for *Apalone,* regulates ciliary development [145].

EMX2, LHX9, LIN54, and ARID3B have known links to sexual development and reproduction, and MEOX2 arises here as candidate for this new putative role. ARID3B may have evolved by developmental systems drift because its new target genes carry out the ancestral function (hypothesis H1A), while EMX2, LHX9, LIN54, and MEOX2 may have evolved by natural selection because their new targets in *Apalone* exhibit differences in their functional annotation from the putative ancestral targets in *Chrysemys* (hypothesis H1B). No evidence was found that the ancestral role of EMX2, LHX9, LIN54, and MEOX2 (i.e., the functions they orchestrate) might have been adopted by another TF (hypothesis H2). Only ARID3B returned primary cilia related terms in both species (Cpi-MPT and Asp-M26 networks), while the primary cilia terms for the other four TFs were found exclusively in *Apalone* networks: all four *Apalone* networks for EMX2 and MEOX2, while solely Asp-M26 for LHX9 and LIN54. This association with male development at cooler temperatures suggests that there may be important sex- or species-specific patterns associated with this organelle, a hypothesis that requires future validation. We now discuss each of these five TFs separately.

***Emx2*** is involved in the formation of cilia [146] which help transduce Wnt signals important for gonadogenesis [74–77], a process EMX2 also mediates in mammals. Our results show EMX2 was more divergent and returned fewer overrepresented terms for Asp*-*M31 (a network lacking strong primary cilia terms) than for all other networks (where primary cilia terms were strongly present), pointing to a potentially reduced EMX2 functionality in Asp-M31, which we hypothesize may prevent warmer temperatures from interfering with proper sexual differentiation of *Apalone* males at ancestrally-feminizing temperatures [19]. Our results may reflect a new role acquired in *Apalone* at late stages where *Emx2* differential transcription could induce differential ciliogenesis, perhaps via natural selection in concert with the evolution of *Meox2.* Indeed, we found evidence of regulatory coevolution for EMX2 and MEOX2. These two TFs clustered tightly within each species (Fig 3D) because their targets overlapped more than any other TFs (by >70%) but also evolved substantially in parallel between *Chrysemys* and *Apalone*. This pattern could occur if they co-regulate the same genes, or if they compete for similar binding sites. This would be possible because TFs can overlap greatly in their active binding of targets yet induce different functional outcomes (via gene expression) due to the combinatorics of other aspects that fine tune regulation and influence the differential usage of shared binding sites [147].

The observed coevolution of *Emx2* and *Meox2* is intriguing because our results would expand the putative roles for ***Meox2***. *Meox2*’s thermosensitive and male-specific expression in turtles [19] combined with its close association with EMX2, a known sexual development gene, render *Meox2* a novel candidate for a role in turtle sexual development whose evolution between *Chrysemys* and *Apalone* may have contributed to transitions in sex determination. MEOX2 returned different biological process terms between turtle species, suggesting a role shift for this TF may have occurred. As this shift implicates the primary cilia, it could affect the thermosensory machinery, a notion supported by the *Drosophila* homologue of MEOX2, *btn,* which mediates responses to noxious temperature [95]. Furthermore, within species, calcium channel terms were overrepresented for MEOX2 at Cpi-MPT but not Cpi-FPT networks, tying this TF to an important known component of epigenetic regulation of TSD gonadal development [21,22]. And intriguingly, *Meox2* represses transcriptional co-activation by β-catenin [95] a key ovarian development gene in TSD turtles [148].

***Lhx9***, another vertebrate gonadal development gene [76,77,79–82] appears to have shifted between our focal TSD and GSD turtles in its location in the regulatory network and in the function of its protein targets, from channels and plasma membrane associated with calcium channels in *Chrysemys*, to nucleus, cytoplasm, and cytoskeleton but not calcium channel terms in *Apalone*. This divergence is of interest because calcium signaling plays an important role in TSD turtles like *Trachemys* [21], and the cytoskeleton affects primary cilia formation and maintenance [84] among other functions. Indeed, *Lhx9* appears prone to developmental shifts despite its putatively critical role in the establishment of the gonad. In *Trachemys*, *Lhx9* is expressed throughout the thermosensitive period but upregulated only in males at stage 15 [20], although it is also present in stage 26 ovaries [149]. Meanwhile, in *Chrysemys*, *Lhx9* is upregulated in males throughout the thermosensitive period. Finally, in *Apalone*, *Lhx9* is upregulated in females or in females developing at ancestrally feminizing (warm) temperature [19]. Such developmental systems drift affects other sexual development genes across turtles and vertebrates [9,24,25].

***Lin54*,** a core subunit of the DREAM/LINC complex which is highly conserved across animals and plants [86,88–91] functions as an activator and repressor by targeting different gene sets [86] and plays roles in development, reproduction in invertebrates [86,87] and perhaps mammals [86] where a paralog of *Lin54*, *Mtl5*, has testis-specific action during spermatocyte meiosis [88]. Interestingly, LIN54 favors binding to autosomes in the soma and influences the X chromosome gene expression indirectly in *C. elegans* [86], where it helps DNA repair [91]. In turtles, we observed significantly overrepresented terms related to development for LIN54 targets in *Chrysemys*, and to primary cilia in *Apalone.* But because LIN54 is related to Wnt signaling (sensed with the help of primary cilia), which is important for gonadogenesis in TSD and GSD turtles [150,151] and other vertebrates [1], our results render *Lin54* an interesting candidate ever-present and primed to help transduce differential Wnt signaling during sexual development.

***Arid3b*** is expressed in numerous cancer types including breast and ovarian cancer [72,152–154] perhaps because it regulates the cell cycle and stem cell genes [152] as it is a member of the LIN28-let-7-ARID3B pathway that promotes cell proliferation [73]. While not itself a member of the DREAM complex, ARID3B binds to E2F and RB family genes [152] which are important members of the DREAM complex [88], and along with LIN54, regulate the mitotic gene *Cdc2* [89,152]. ARID3B is also expressed in the Leydig cells [72], and binds to *Wnt1* [73]. Importantly, our results showed that ARID3B was most differential between *Chrysemys* networks, where it targets genes related to channel activity and membrane transport at Cpi*-*FPT in agreement with the reported regulation of TSD female development via the phosphorylation of STAT3 mediated by calcium channels, which represses *Kdm6b* expression and consequently, the epigenetic activation of *Dmrt1* and downstream male-differentiation genes [22]. Our observations render ARID3B an important upstream candidate for male and female development in *Chrysemys* with putative opposite action to MEOX2 which exhibited overrepresentation of calcium channel terms at Cpi-MPT but not Cpi-FPT networks in this TSD turtle.

### Evolution of *Dhh*, a known candidate gene, is also linked to the turnover in sex determination associated to primary cilia

We previously identified *Dhh* (Desert Hedgehog Signaling Molecule) as a gene with sex-specific expression that switched between *Chrysemys* and *Apalone* [19]. *Dhh* is upregulated at FPT (31°C) during the thermosensitive period in *Chrysemys*, and in males or at 26°C in *Apalone* at the same stages (15–22) [19]. In contrast, *Dhh* was not expressed during a similar time window in *Trachemys* (stages 15–19 and 21) [20]. *Dhh* is involved in mammalian testis development and upregulated in male mice during stages e11.6-e12.0 which correspond to turtle stages 18–21 [20,155,156]. *Apalone’s* transcriptional pattern agrees with mammalian *Dhh* expression and its role in testis development, suggesting an evolutionary shift between *Apalone* and *Chrysemys* lineages in *Dhh* regulation. Our qualitative results suggest that the TFs NFIA and CTCF may have increased their targeting of *Dhh* in *Chrysemys* relative to *Apalone*, and 17 other TFs may have increased targeting of *Dhh* in *Apalone* relative to *Chrysemys,* including LHX9, MEOX2, EMX2, and ARID3B related to primary cilia, plus BARHL2, LHX2, ISL2, ALX1, HESX1, LHX8, ELF5, HIF1A, HEY2, MNT, VAX1, BHLHE40, and MSX1 (implicated in female pathways via active male downregulation [1]).

Hedgehog signaling is dependent on primary cilia in vertebrates, better characterized for hedgehog genes *Shh* and *Ihh* [157], but also *Dhh* [158]. Specifically, hedgehog signaling components were found in primary cilia of immature Leydig cells [158,159], whose differentiation is induced by DHH signaling, thus rendering DHH signaling via primary cilia a potential regulator of Leydig cell recruitment and or differentiation [158–160]. DHH signaling is also present in mouse ovary shortly after birth where it participates in theca cell differentiation [161], pointing to a general role in specification of steroidogenic cells in the gonad. Furthermore, a direct link between DHH signaling and primary cilia via a Type II non-canonical cilia signaling mechanism was detected in the developing mouse heart [162].

### Novel *Primary Cilia Integration* hypothesis extends the calcium and redox (CaRe) sex determination model

Primary cilia are antennae-like organelles, now recognized as essential for the perception and transduction of signals in most cell types, whose disfunction is linked to numerous diseases, including hypogonadism and genitourinary disorders of development (reviewed in [32]). The primary cilium consists of a basal body made up of the centriole (which moonlights as the mitotic spindle), a transition zone (the cilium gateway), and an axoneme typically made up of nine microtubule doublets [32,74]. Proteins are moved up and down the axoneme via anterograde (IFT-A, kinesin) and retrograde (IFT-B, dynein) transport. The ciliary membrane can contain numerous proteins including TRP channels, which are important for relaying Ca^2+^, to communicate environmental changes such as temperature, mechanical force, and other signals, and is key to the primary cilia’s ability to integrate environmental inputs to the cell [163–165]. Primary cilia are also involved in relaying numerous signaling pathways beyond hedgehog and Wnt, including GPCR, TGF-B/BMP, NF-kB, among several others [32,157,166–169]. Thus, primary cilia are uniquely suited to integrate environmental cues into developmental outputs, such as is essential for developmental plasticity.

Our findings suggest a link between primary cilia and turtle sexual development, expanding previous reports showing they play a critical role in the development of the urogenital ridge [142], Wolffian ducts (mediated by DHH signaling) [143], and somatic and germline gonadal components in mammals [142,144] and perhaps also in *Paralichthys olivaceus*, a fish with a thermosensitive XY system [170]. Namely, TFs with overrepresented terms related to the primary cilia present in *Chrysemys* and *Apalone*, include ARID3B, EMX2, LHX9, LIN54, and MEOX2 described above, plus DLX2, HIC2, HMBOX1, HOXD3, LHX2, MSX1, NFIA, SHOX, SHOX2, SMAD4, SOX10, and ZEB1, of which LHX2 [115,116], MSX1 [118–120], SMAD4 [121], and SOX10 [122] have known ties to gonadal development. Because primary cilia are organelles found in nearly all vertebrate cell types that help cells understand the context of cues from the environment or signaling pathways, including Wnt signaling [171] and hedgehog signaling [157], here we propose a new testable hypothesis based on our results and basic primary cilia biology, that integrates and expands upon the calcium and redox (CaRe) model of sex determination [21]. The CaRe model posits that thermosensitive cytoplasmic calcium and mitochondrial redox signaling interactions activate or repress male- and female-specific developmental pathways [21]. And we note that importantly, primary cilia are linked to both reactive oxygen species (ROS) and calcium signaling.

**Fig 4:**
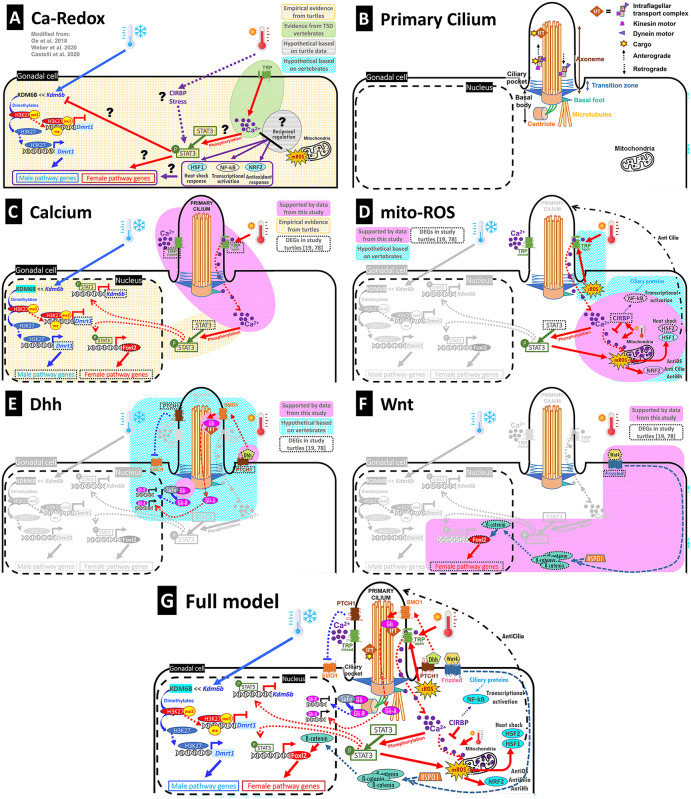
*Primary cilia integration (PCI)* hypothesis of temperature-dependent sex determination and differentiation. This hypothetical model is informed by findings in this study from *Chrysemys picta* and *Apalone spinifera* and from literature on other TSD turtles, and TSD and GSD vertebrates (see text for further details). Some components of this working model may be cell type-specific and stage-specific, and some may have pleiotropic effects in other functions and tissues that do not invalidate their potential participation in turtle sexual development as hypothesized here. A) Previous Calcium-Redox model where thermosensitive calcium-redox signaling activates or represses sexual development pathways [21]. B) Primary cilium structure [32,74]. C) PCI-hypothesized calcium signaling mediated through the primary cilium with reported effect of calcium on sex determination [22]. D) PCI-hypothesized calcium signaling mediated through the primary cilium with potential cross talk to mitochondrial reactive oxygen species [187,188] and activation of known associated pathways [189,190]. E) PCI-hypothesized thermosensitive hedgehog signaling mediated through the primary cilium, of which DHH is involved in gonadal development [158–160], and our hypothesis testing results suggest this signaling pathway might be differentially regulated between *Chrysemys* and *Apalone*, including its Gli proteins component. F) Thermosensitive Wnt signaling potentially mediated by the primary cilium with known influence on sex determination pathways [74–77], whose components are differentially expressed in developing female *Chrysemys* and *Apalone* [Radhakrishnan et al. 2017; Gessler et al. 2023. G) Full *Primary Cilia Integration* model. TRP channels would open at warm temperatures, allowing Ca^2+^ influx into the primary cilium. This Ca^2+^ could be relayed to the cytosol and transported to the mitochondria, potentially contributing to the production of reactive oxygen species (ROS), which would alter the CaRe status of the cell. At lower temperatures, TRP channels would be closed, blocking entry of Ca^2+^. CIRBP would inhibit movement of Ca^2+^ into the mitochondria and may help modulate the CaRe state. CaRe crosstalk may influence signaling pathways like HSF1 (in which HFS2 participates [183]), NF-kB, and NRF2 [21], which have known ties to the primary cilium [189,190], of which NF-kB signaling is mediated by CIRBP [190]. Under the PCI, when high temperatures raise Ca^2+^ levels, STAT3 is phosphorylated by a still unknown factor [perhaps by JAK family kinases [191]]. In TSD turtles, pSTAT3 is known to inhibit transcription of *Kdm6b*, a histone demethylase [22]. When STAT3 is unphosphorylated at cooler temperatures, *Kdm6b* is expressed and can activate *Dmrt1* through demethylation of H3K27me3, driving expression of male pathway genes. Failure to produce KDM6B protein results in retention of silencing chromatin marks (H3K27me3) at the *Dmrt1* promoter, inhibiting expression of male pathway genes [22]. Furthermore, pSTAT3 binds to the *Foxl2* promoter, driving downstream expression of female pathway genes [173]. *Foxl2* expression may be further enhanced by the accumulation of β-catenin in response to Wnt signaling which is stabilized by RSPO1 [192]. Wnt signaling can be mediated by the primary cilium [32] and is thermosensitive in TSD turtles [193]. DHH, another signaling pathway important for sexual development and dependent on the primary cilium [158], may be temperature sensitive and evolutionarily labile [194]. When activated, DHH ligands binds to PTCH1 receptors which allows activation of SMO1, a protein that converts GLI transcription factors into active forms that can activate downstream target genes [98,162].

Our *Primary Cilia Integration* hypothesis (Fig 4) proposes that primary cilia might be important antennae relaying environmental cues and integrating them via the signaling pathways they mediate [32,163–165] to help guide sex determination and differentiation in TSD turtles. Warmer temperatures would mediate calcium signaling through TRP channels present in ciliary membranes [163,164,172] and TRP genes are already implicated in TSD biology [21,22,85]. Calcium fluxes through TRP channels that open at warmer temperature induce the documented phosphorylation of STAT3 which is demonstrated to inhibit *Kdm6b* expression in TSD turtles, thus favoring female developmental pathways [9,22,173]. Additionally primary cilia are known in other vertebrates to transduce Wnt signaling [83,168,174], hedgehog signaling [143,158,162], NF-kB signaling [169], and are negatively regulated by NRF2 signaling [175,176], all pathways with reported links to sex determination [21]. Wnt’s and hedgehog’s signaling role in vertebrate gonadal development is well documented: (a) Wnt canonical signaling is linked to female development [77,148,150,177], increases under female producing temperatures in the snapping turtle *Chelydra serpentina* [150], and failure to inhibit it in humans disrupts male development [178]; (b) hedgehog canonical signaling is linked to male development and is entirely dependent on the primary cilium [143,157–159]; and (c) Wnt and hedgehog signaling can be mutually antagonistic [179] consistent with the mutual inhibition required for alterative commitment to male or female developmental fate [1]. NRF2, a TF overrepresented in our study, is important for germ cell proliferation, survival, and spermatogenesis in vertebrates by combating mitochondrial ROS in gonads [180,181]. HSF2, a TF differentially expressed in turtles [Gessler et al. 2023] and involved in vertebrate spermatogenesis [182], interacts with HSF1 during heat shock and oxidative stress response in vertebrates [183,184]. And the TF NF-kB, which is differentially transcribed in turtles [19,78], also participates in vertebrate gonadal differentiation [185,186]. Research is warranted to determine the cells and developmental stages when specific steps or components of this model take place or are active, in order to functionally validate, rule out, or modify this hypothesis.

The PCI expanded model reveals several new questions to guide future studies in turtles, and multiple aspects of the model must be functionally validated, such as exploring the thermosensitivity of hedgehog, identifying the agent which phosphorylates STAT3, and characterizing the makeup of primary cilia membranes. The evolutionary changes revealed by our analyses as described earlier suggest that important modifications might have occurred at several levels in *Apalone* compared to *Chrysemys*, including in the rate of ciliogenesis, in the morphology and composition of the primary cilia, and in the location of ciliary proteins (ciliary versus extraciliary) which can affect their functions (altering cell cycle regulation, cytoskeletal regulation, and trafficking) [195]. Additionally, the species-specific role of each of the candidate TFs identified here must be tested.

Another important question is how do sex chromosomes interface with the primary cilia as would be predicted if primary cilia underlie the evolution of vertebrate sex determination? This question is challenging because the sex-determining genes of turtles with sex chromosomes have not been identified, such that it is unclear whether *Apalone*’s GSD system is controlled by a dominant W factor or by the dosage of a recessive Z factor, but other sex-linked genes involved in gonadal development are known in turtles. For instance, *Wt1*, the Wilm’s tumor protein 1 gene involved in the development of the bipotential gonad and later testes [196] or ovaries depending on the spliceoform [197], causes Wilm’s tumors which are associated with primary cilia disfunction [198]. And intriguingly, *Wt1* is linked to the sex chromosomes in *Glyptemys insculpta* and *Siebenrockiella crassicollis* turtles, two species with independently evolved XY systems [199]. Likewise, *Dmrt1*, a testis development gene tied to testicular germ cell cancer which is also associated with primary cilia disfunction [200], is linked to the sex chromosomes of *Staurotypus triporcatus* turtles, another lineage with an independently evolved XY system [199]. Further, the steroidogenic factor 1 gene *Sf1*, a target and partner of *Wt1* during gonadal development [192], is tied to metabolic homeostasis [201] that primary cilia mediate [202], and is linked to the ZW sex chromosomes of *Apalone spinifera* softshell turtles [203]. Emerging resources, such as the development of turtle organoids [204], will provide an excellent functional genomics resource in which to investigate the potential role of primary cilia in sensing the environment during gonadal sex determination and differentiation for TSD species. Indeed, previous studies have examined the effects of primary cilia in mammary organoids [105,109].

## Conclusions

We generated gene-transcription factor sexual development networks for two turtle species, *Chrysemys picta* and *Apalone spinifera*. While generalized in scope due to sample pooling, the characteristics of these networks are consistent with previously reported biology of sexual development. These networks represent mechanistic hypothesis to inform future investigations into the evolution of sex determination in turtles and vertebrates. Our results support the following conclusions that can drive future targeted research efforts: (1) There may be a large degree of conservation in transcription factor hubs between *Chrysemys* and *Apalone*, consistent with the prevailing understanding of a high degree of conservation in elements of vertebrate sex determination networks and evo-devo toolkit hypotheses, but that warrant further research with larger sampling to rule out the alternative that differences exist in these components that pass undetected in our study. (2) While the TF hubs appeared conserved, the targets of shared hubs often were not. In some cases, these varying targets converged on similar functional annotations, suggesting a role for developmental systems drift. In other cases, target genes significantly differed in their functional annotations suggesting a possibility of natural selection or genetic drift to be at play. (3) We identified one TF with conserved targets (perhaps ancestral) that may have undergone developmental systems drift to target additional genes targets during GSD evolution or alternatively, an example of a dozen TFs that may have converged in TSD to regulate the targets of the conserved TF. (4) Several candidate TFs were detected that are of interest as they have known roles in mammalian sexual development and our analysis expands their role to sex determination in reptiles. (5) Qualitative results identified predicted regulatory changes in *Dhh* that could underpin its male-to-female shift in gene expression previously observed between *Apalone* and *Chrysemys*. (6) Finally, our findings suggest that primary cilia might be tightly linked to sexual development in both TSD and GSD species. Thus, we propose a potential role for primary cilia as the sentinels of environmental signaling in TSD, explicitly linking them to this function while expanding upon the CaRe Hypothesis, and tying them to transitions in sex determination for the first time. Given the ability of the primary cilium to interface with the environment and with so many signaling pathways (several with known ties to sexual development), it is tempting to hypothesize that they could underly the evolutionary diversity observed in vertebrate sex determination more broadly.

Further research is warranted to test these hypotheses, including comparative proteomics of primary cilia in developing gonads of TSD and GSD species to elucidate finer details of their compositional dynamics during cell differentiation at various embryonic stages of sexual development. This approach is still missing both in our general understanding of primary cilia function and in their role in development and disease [32], but will also help decipher the role of primary cilia in the molecular and cellular evolution of plasticity and canalization.

## Supporting information

S1 File Supporting Information. (ZIP)

## Abbreviations

CaRe: calcium redox
DAG: directed acyclic graph
ESD: environmental sex determination
FPT: female producing temperature
GRN: gene regulatory network
GSD: genotypic sex determination
MPT: male producing temperature
Mya: million years ago
PC: principal components
ROS: reactive oxygen species
SDM: sex determination mechanism
TF: transcription factor
TFBS: transcription factor binding site
TSD: temperature-dependent sex determination
